# Effects of Information Length and Implementation Intentions on Adherence to Weight Management Strategies: Experimental Study

**DOI:** 10.2196/65260

**Published:** 2025-08-08

**Authors:** Khaleda Ahmadyar, Joanna Szypula, Angeliki Bogosian, Katy Tapper

**Affiliations:** 1 School of Health and Medical Sciences, City St George's, University of London, Northampton Square, London, EC1V 0HB, United Kingdom, 44 020 7040 5060

**Keywords:** weight management, behaviour change, adherence, implementation intention, experimental study, weight management strategy, weight loss, weight, strategy, engagement, understanding, intervention, efficacy, brief weight management, linear regression, mobile phone

## Abstract

**Background:**

Adherence to weight management strategies may be undermined where lengthy strategy explanations limit engagement and understanding, weakening intervention efficacy. By contrast, implementation intentions have been shown to promote adherence across various health behaviors.

**Objective:**

This study aimed to investigate the impact of explanation length and implementation intentions on adherence to brief weight management strategies.

**Methods:**

Participants (N=200) with a BMI above 25 and an interest in losing weight were recruited from a commercial digital weight management service provider. Participants received information about 1 of 4 weight management strategies on a smartphone app in either a brief or detailed format and were asked to plan their use of the strategy with implementation intentions or were given tips on strategy use. Participants received daily prompts over a 2-week period to report whether they used their assigned strategy. Proposed moderators (need for cognition and planning skills) were measured at baseline.

**Results:**

Strategy adherence was greater with brief information (mean 74%, SD 23%) compared with detailed information (mean 69%, SD 23%); however, this small effect size (Cohen *d*=0.24) was not statistically significant (*P*=.13). There was no moderation by need for cognition (*P*=.25). Adherence did not differ significantly between implementation intentions (mean 71%, SD 27%) and tips (mean 72%, SD 21%; *P*=.73); however, there was moderation by planning skills (*P*=.04). As predicted, adherence was greater with implementation intentions compared with tips among those with poorer planning skills.

**Conclusions:**

Shorter explanation length and implementation intentions (in poorer planners) may enhance adherence to brief weight management strategies, and further investigation is required to confirm these effects.

## Introduction

Successful weight loss and weight loss maintenance require adjustments to food intake and physical activity; the key behaviors targeted by most weight management interventions [1]. These interventions include a variety of behavioral and psychological strategies, ranging from calorie counting [2] to mindful eating [3] and intermittent fasting [4]. While the efficacy of many of these interventions is well-established [5], their impact on weight management remains contingent upon individual adherence. However, evidence shows that adherence is often suboptimal and can be as low as 10%, undermining intervention effectiveness [6]. There is also some evidence that adherence is lower in individuals with lower socioeconomic status [7], which may contribute to health inequalities. Therefore, it is crucial to explore methods by which adherence can be increased to maximize the potential benefits of interventions.

One important factor to consider is the way the information is presented. Communication in health promotion is crucial [8], and effective communication involves tailoring messages for the intended audience [9]. The information-motivation-strategy model [10] states that one of the main reasons individuals do not adhere to behavior change advice is that they are not given adequate, understandable information. One aspect that could influence understanding is the length of the written information provided. Although longer and more detailed information may enhance understanding, it is possible that longer material reduces engagement, which in turn limits understanding and implementation. By contrast, briefer information may enhance engagement, leading to increased understanding and implementation.

This idea is supported by research on attentional processes, which play a critical role in how individuals process information [11]. In recent years, there has been a decline in attention span, attributed in part to increased information overload due to the rapid rise of digital technology [12]. Yeykelis et al [13] found that individuals tend to switch between different types of web-based content as frequently as every 19 seconds, and 75% of all content is typically viewed for less than 1 minute. This type of media multitasking is associated with worse performance on cognitive tasks requiring long-term attention [14]. This trend emphasizes the importance of capturing and maintaining individuals’ attention to enhance their engagement with the content of weight management strategies. Given the reduction in attention span, individuals may be more likely to engage with shorter, more focused content, while longer information may lead to cognitive overload and skim-reading or disengagement entirely.

Nevertheless, preference for longer versus shorter information may vary from person to person. For example, the Need for Cognition Scale (NCS) [15] assesses individuals’ inclination to engage in and enjoy cognitive activities. Therefore, those with a higher need for cognition may be more willing to engage with lengthier written material and more interested in learning the rationale behind a particular weight management strategy. By contrast, those with a low need for cognition may be put off by lengthier material, preferring information that is more succinct and to the point. This hypothesis has been partially supported by Williams-Piehota et al [16], who found that women who were high in need for cognition were more likely to follow more detailed (compared with more succinct) mammography advice. However, the format made no difference for those who were low in need for cognition.

Another key factor that may influence adherence is difficulty translating intentions into action, that is, the intention-behavior gap [17]. Many individuals may understand and be motivated to use strategy information, yet still fail to implement it. The Rubicon model of action phases [18] posits 2 key phases of goal pursuit: a motivational (predecisional) phase when the individual forms the intention to perform the behavior, and a volitional (postdecisional) phase when the behavior is implemented. The theory suggests that behavior change can be promoted by targeting motivation in the predecisional phase and implementation of the behavior in the postdecisional phase [19]. A powerful strategy for the latter phase is the formation of an implementation intention, which involves the development of a specific plan of action in the form of an if-then statement, for example, “If situation X is encountered, then I will initiate behavior Y” [20]. Systematic reviews have found implementation intentions to be effective in improving general goal attainment [21], and adherence to a range of health behaviors such as healthy eating [22,23], physical activity [24-26], and smoking cessation [27]. The use of implementation intentions in weight loss interventions has also been associated with greater weight loss [28,29]. However, findings are mixed; Benyamini et al [30] and Hayes et al [31] found that implementation intentions resulted in similar weight loss outcomes as simple goal intentions. In addition, Knäuper et al [32] found that the addition of implementation intentions to the National Institutes of Health–developed Diabetes Prevention Program did not result in greater weight loss.

This discrepancy may be due to the different ways implementation intentions are used across studies. A range of variables may moderate the effects of implementation intentions, including the quantity [33] and specificity of the intentions [34]. Individual differences in self-regulation may be another potential moderator; whether implementation intentions are helpful for an individual could be contingent upon their proficiency in planning skills. Allan et al [35] instructed participants to complete a web-based food diary to monitor their snack intake, and half were also instructed to generate an implementation intention to help them achieve this goal. The implementation intention intervention was significantly associated with higher completion rates in poorer planners, but not in skilled planners, suggesting that adherence can be enhanced by tailoring interventions to individuals’ planning skill abilities.

In light of the above, this study investigated whether adherence to brief weight management strategies over a 2-week period is influenced by the length of strategy information and the use of implementation intentions. The aims were to (1) explore the effect of information length on adherence, and whether this differs depending on need for cognition, and (2) examine the effect of implementation intentions on adherence, and whether this differs depending on planning skills. Given an absence of previous research, we did not formulate any confirmatory hypotheses related to the first aim. However, for the second aim, we predicted that use of implementation intentions would increase adherence, and that this increase would be larger for those with poorer planning skills.

## Methods

### Sample Size

A minimum sample size of 128 participants was calculated on G*Power based on a medium effect size, 80% power, and 5% α for a 2×2 ANOVA. To account for participant attrition and exclusions, and the testing of exploratory hypotheses, the target sample size was set at 200 participants.

### Participants

Participants were patients recruited from Oviva, a digital commercial weight-management service provider. Individuals may access the service via self-referral or general practitioner referral, depending on the program they would like to enroll in. The cost of the service is covered by the UK National Health Service. Individuals on the 9-month Diabetes Prevention Program, 12-week tier 2 weight management, and 12-week diabetes structured education programs were invited to take part in the study by email. To prevent the study from interfering with program engagement, participants were invited when they had completed the program or were close to completion. Additional eligibility criteria were age 18 years or older, access to a smartphone, a BMI of more than 25, an interest in losing weight or avoiding weight gain, and not on a meal replacement diet.

### Ethical Considerations

The study received ethical approval from the City, University of London Psychology Department Research Ethics Committee (ETH2223-2482). The method and analysis strategy were preregistered with the Open Science Framework [36].

Participants provided informed consent (Multimedia Appendix 1) before enrolling in the study. Study data were anonymized to ensure that individual participants could not be identified. Participants received Amazon vouchers worth up to £20 (approximately US $25) based on participation duration, with an extra £5 (approximately US $6.25) for completing an optional qualitative part of the study.

### Study Design

The study used a 2×2×4 between-groups experimental design. Participants were randomly assigned to 1 of the 16 groups, which varied by information length (short or long), implementation intentions (present or absent), and strategy content (sensory eating, attending to fullness, eating vegetables first, or increasing physical activity). The dependent variable was the percentage of days participants adhered to their assigned strategy.

The study also examined whether higher NCS scores were associated with a preference for longer information, and whether use of implementation intentions was associated with increased strategy automaticity during the 2-week period. In addition, the study investigated whether briefer, more lay-friendly measures may be adequate substitutes for the longer, standardized measures of need for cognition and planning skills. This was considered important since although these measures may prove useful for increased personalization of interventions, their length may make them too burdensome and impractical for digital interventions, especially where multiple characteristics are being assessed.

Effects of information length on ease of understanding and remembering strategy content, as well as memory of strategy rationale, were also investigated. In addition, differences in adherence to mental and physical strategies were explored. To identify potential differences in adherence between different types of mindful eating strategies, 2 distinct mindful eating strategies were examined (attending to feelings of fullness versus attending to sensory properties of food). In addition, 2 further evidence-based, nonmindfulness weight management strategies (eating vegetables before other food groups and performing physical activity immediately following a meal) were included in the study to identify any differences between strategies that require mental effort as opposed to those that require physical effort. Finally, the research also aimed to gain qualitative insights into participants’ views on the weight management strategies and their experiences during the study.

### Experimental Manipulation

#### Strategy Content

Evidence-based written information was provided for 1 of the 4 brief weight management strategies: paying attention to the sensory properties of food while eating [37], paying attention to feelings of fullness while eating [38], eating vegetables or salad before the rest of the meal [39], and doing 5 minutes of physical activity following a meal [40]. The information provided the rationale behind the strategy as well as instructions on how to practice it (refer to Multimedia Appendix 2 for full strategy information).

#### Information Length

The strategy information was either presented in a short format (approximately 70‐100 words) with a focus on action (eg, pay attention to the taste and texture of food in your mouth) or a long format (approximately 600‐700 words) with a focus on outcome (eg, how to slow down your eating) and additional detail on the strategy rationale.

#### Implementation Intentions

Participants were either presented with planning prompts to help them form implementation intentions (present), or they received tips on strategy use (absent). The planning prompts involved first indicating when they would use the strategy (eg, If I am eating breakfast), followed by how they would use it (eg, then I will keep reminding myself to notice the taste, texture, and temperature of the food). The same content was presented in the tips condition in the form of tips on strategy use (eg, when you are eating a meal or snack, keep reminding yourself to notice the taste, texture, and temperature of the food).

It is important to note that we compared implementation intentions with the type of standard behavioral advice typically used by health care providers, such as Oviva, which consisted of information, followed by a series of “tips.” Like implementation intentions, these tips also linked an environmental cue with a specific action. However, unlike implementation intentions, they did not follow an “if-then” format, had slightly less cue specificity, and were received passively by the participant, rather than being actively selected. We were interested in whether this type of “gold standard” implementation intentions would outperform the type of behavioral advice already being used by the health care provider.

### Materials

The study was delivered on the Avicenna Research (formerly Ethica Data) smartphone app (Avicenna Research Inc) [41]. A baseline survey, a schedule of 14 daily surveys, and a follow-up survey were triggered upon enrollment. Participants were notified to complete the surveys via phone notifications.

### Measures

#### Baseline Measures

##### Demographics

Participants indicated their age, gender, ethnicity, and education level.

##### Height and Weight

Self-report measures were provided in kilograms or pounds and centimeters or feet and inches.

##### Weight Loss Intentions

Participants responded to *“*Which of the following best describes you?” with “I’m trying to lose some weight,” “I’m not trying to lose weight but I’m trying to avoid gaining weight,” or “I’m not currently trying to lose weight.”

##### Planning Skills

Planning skills were assessed using 10 items from the “goal setting” subscale of the Short-form Self-Regulation Questionnaire (SSRQ) [42]. Items were rated on a 5-point Likert scale ranging from 1 (strongly disagree) to 5 (strongly agree). The total SSRQ score was computed by summing the 10 items; higher scores indicated greater planning skills.

##### Alternative Measure of Planning Skills

Participants responded to “Which of the following best describes you?*”* with “I’m good at making plans and sticking to them. If I set myself a goal, I’ll spend time figuring out exactly how to reach it. If I’m not making good progress toward a goal, I’ll go back to my plans and think again;” or “I struggle to make plans and stick to them. I often find myself forgetting to do things I’d planned to do or getting distracted with other things.” This 1-item measure was developed by simplifying and consolidating the items on the SSRQ using more accessible, lay-friendly language.

##### Need for Cognition

Need for cognition was assessed using the short form of the NCS [43], which consisted of 6 items, each rated on a 5-point Likert scale ranging from 1 (extremely uncharacteristic of me) to 5 (extremely characteristic of me). A mean NCS score was computed from the 6 ratings, with higher scores indicating greater need for cognition.

##### Alternative Measure of Need for Cognition

Participants responded to “Which of the following best describes you?*”* with “If a doctor gives me advice, I like to understand the reasoning behind that advice. I’ll ask questions or search the internet until I feel I really understand the issue;” or “If a doctor gives me advice, I’m usually happy to simply take that advice. I don’t feel the need for lengthy explanations and justifications.” This 1-item measure was developed by simplifying and consolidating the items on the NCS using more accessible, lay-friendly language.

### Daily Measures

#### Adherence

Each day, for 14 days, participants were asked, “Did you use the strategy today?*”* with response options “Yes, I used it at least once,” “No, I forgot to use it,” or “No, I didn’t have time or didn’t use it for another reason.” Adherence was calculated as the percentage of days participants indicated they used the strategy out of the total number of daily surveys they completed.

### Follow-Up Measures

#### Ease of Understanding and Remembering Strategy Information

Participants responded to “How easy was it to understand the instructions for the strategy?” and “How easy was it to remember the information you were given about the strategy over the 2-week period?” on a 100 mm visual analog scale anchored from 0 (very difficult) to 100 (very easy).

#### Views on Length of Strategy

Participants were asked, “When we gave you the strategy, we also explained why it might be helpful. Was this explanation…*”* with response options “too short?,” “about right?,” or “too long?”

#### Automaticity of Strategy Use

The 4-item Self-Report Behavioral Automaticity Index (SRBAI) [44] assessed whether the assigned strategy became a habit over the 2-week period. Items were rated on a 7-point scale ranging from 1 (strongly agree) to 7 (strongly disagree). The 4 ratings were reverse-coded and summed to form a total score with higher scores indicating higher levels of automaticity.

#### Memory of Strategy Rationale

Four multiple-choice questions, one relating to each of the strategies, were used to assess participants’ knowledge of the rationale behind each strategy (Multimedia Appendix 3).

#### Qualitative Questions

Furthermore, 4 optional open-ended questions were administered to explore participants’ experiences during the study. Refer to Multimedia Appendix 4 for details of the qualitative aspect of the study.

### Procedure

Data collection took place between August and November 2023. Eligible Oviva patients were emailed the study advert, and interested participants first completed a screening survey on Qualtrics to confirm eligibility. Participants then completed the consent form and were given enrollment instructions. Once enrolled, participants completed the baseline measures and were presented with their assigned strategy, followed by planning prompts or tips on strategy use. The short and long versions of each of the 4 strategies were independently paired with the corresponding planning prompts and tips and presented in separate sections of the survey. This created 16 unique combinations, resulting in 16 sections. Participants were randomly assigned to view one of these sections using simple randomization via the Avicenna Research app. It took approximately 30 minutes to complete baseline measures and read the strategy information. Participants then selected their preferred notification time (between 6 PM and 11 PM) for the daily surveys and were notified at their chosen time each day over the next 14 days to complete the surveys, which took less than a minute. To avoid participants mistakenly recording answers for the wrong day, each daily survey expired within 24 hours.

At 9 AM on the day following the last daily survey, participants were prompted to complete the follow-up measures. This took approximately 30 minutes. Following this, participants were given the option to complete the qualitative survey, which took an additional 30 minutes. Participants were then provided with a written debrief, and payment vouchers were issued via email.

### Data Analysis

Data were analyzed in the IBM SPSS statistical analysis package (version 28). All analyses were subject to bootstrapping at 5000 resamples. Linear regression models were used to test the effects of information length and implementation intentions on strategy adherence. The moderating effects of NCS and SSRQ scores were examined using multiple regression models (model 1) via the PROCESS macro [45]. The association between NCS score and participants’ preference for information length was explored with an ordinal logistic regression model. An independent *t* test examined the effect of implementation intentions on SRBAI scores.

Point-biserial correlation tests were used to test the association between the 2 need for cognition measures and the association between the 2 planning skills measures. The moderating effect of the alternative need for cognition measure on the association between information length and adherence was explored using ANOVA, as was the moderating effect of the alternative planning skills measure on the association between implementation intentions and adherence. ANOVA was also used to explore the differences in adherence across the 4 strategies.

The effect of information length on ease of understanding and remembering strategy information was explored using linear regression, and PROCESS model 1 tested the moderating effect of NCS score. A logistic regression model evaluated the effect of information length on the likelihood that participants remember the rationale for the strategy they were assigned to, with NCS score entered as a moderator. Qualitative data were analyzed using content analysis.

## Results

### Participant Characteristics

In total, 200 participants enrolled in the study. Participants’ age ranged from 23 to 79 years, with a mean of 52 (SD 11.2) years. The sample consisted of 63% (126/199) women. BMI ranged from 25.3 to 64.9 kg/m^2^ with a mean of 35.5 (SD 7.3) kg/m^2^. Most participants (155/197, 78%) were White, 12% (23/197) were Asian or Asian British, 5% (10/197) were Black, African, Caribbean, or Black British, 4% (7/197) were Mixed or from multiple ethnic groups, and 1% (2/197) were from other ethnic groups. Half of the sample (102/200, 51%) had an undergraduate degree or higher, 22% (43/200) were educated to General Certificate of Secondary Education (GCSE) level, 11% (22/200) had a Business and Technology Education Council (BTEC) qualification, 11% (21/200) had Advanced levels (A-levels), 4% (7/200) had no formal education, and 3% (5/200) had another form of qualification. In the UK education system, students typically undertake GCSEs at age 16. A-levels are typically taken at age 18 as a qualification for university entry, while BTEC qualifications provide vocational training.

**Figure 1.**
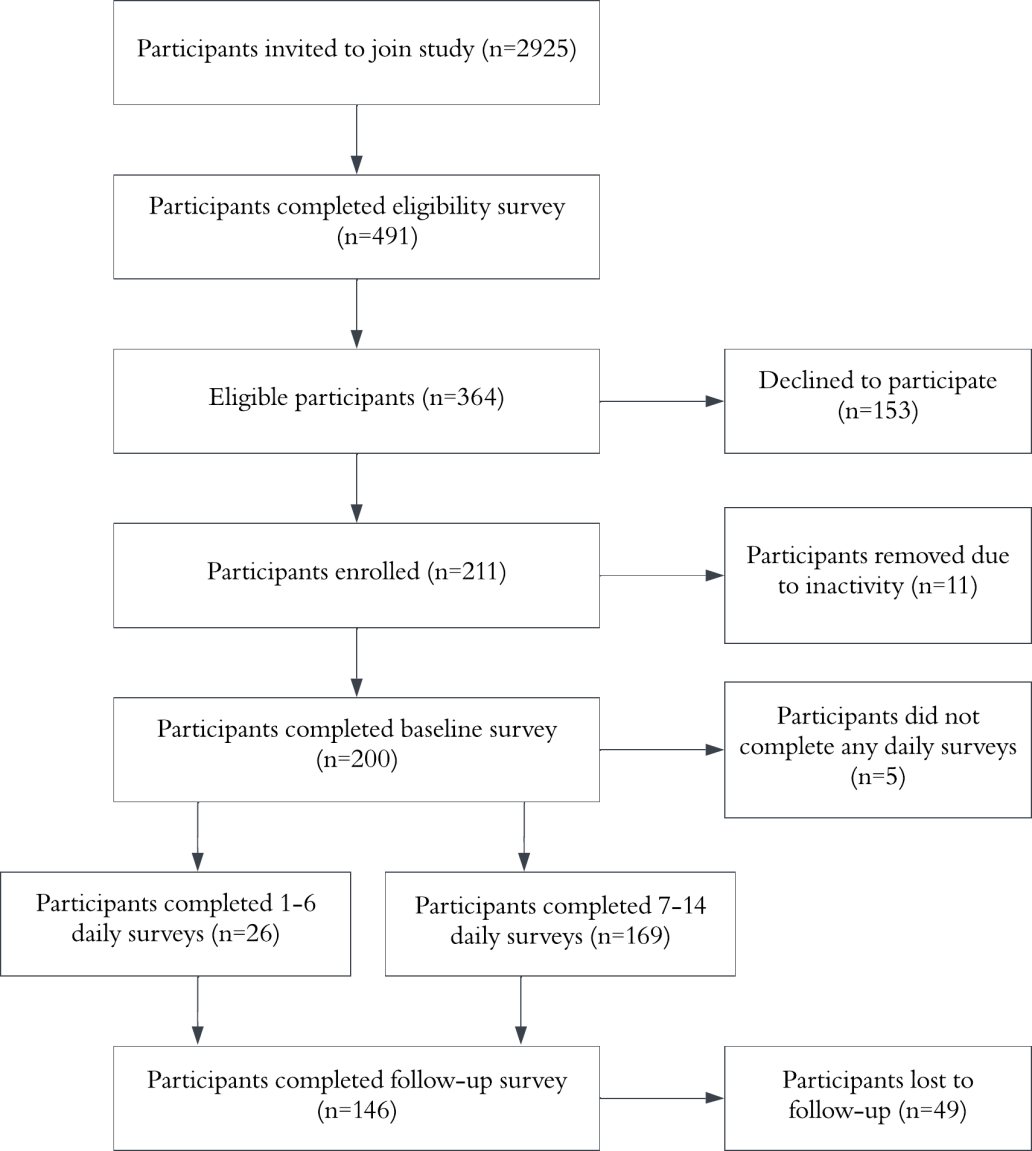
Flow chart of participants through the study.

Figure 1 presents the flow of participants through the study. As per the preregistration, only participants with data for 7 or more daily surveys were included in the main analyses, exploring the effects of information length and implementation intentions (n=169). Analyses relating to follow-up measures were restricted to participants who completed the follow-up survey (n=140). For all other analyses, the full sample was used (N=200). Participant characteristics of those included in the main analyses (n=169) were well-matched across conditions (Table 1).

**Table 1.** Participant characteristics as a function of condition.

Characteristic	Implementation intentions (n=80)	Tips (n=89)	Short format (n=85)	Long format (n=84)	All (n=169)
Age, mean (SD)	53 (12)	51 (10)	51 (11)	53 (11)	52 (11)
Gender (women), n (%)	52 (66)^a^	58 (65)	53 (63)^b^	57 (68)	110 (65)^c^
BMI, mean (SD)	35.3 (6.5)	35.9 (8.3)	35.7 (7.2)	35.6 (7.8)	35.6 (7.5)
Education, n (%)					
No formal education	2 (3)	4 (5)	2 (2)	4 (5)	6 (4)
GCSEs^i^, O-levels, or equivalent	18 (23)	20 (23)	17 (20)	21 (25)	38 (23)
A-levels or equivalent	7 (9)	7 (8)	6 (7)	8 (10)	14 (8)
BTEC^e^ or equivalent	10 (13)	9 (10)	9 (11)	10 (12)	19 (11)
Undergraduate degree or equivalent	23 (29)	33 (37)	28 (33)	28 (33)	56 (33)
Master’s degree or equivalent	16 (20)	15 (17)	18 (21)	13 (16)	31 (18)
Doctoral degree or equivalent	1 (1)	0 (0)	1 (1)	0 (0)	1 (1)
Other	3 (4)	1 (1)	4 (5)	0 (0)	4 (2)
Ethnicity^f^, n (%)					
Arab	0 (0)	0 (0)	0 (0)	0 (0)	0 (0)
Asian or Asian British	6 (8)	11 (13)	10 (12)	7 (8)	17 (10)
Black, Black British, Caribbean, or African	3 (4)	4 (5)	5 (6)	2 (2)	7 (4)
Mixed or multiple ethnic groups	2 (3)	2 (2)	2 (2)	2 (2)	4 (2)
White	67 (85)	70 (81)	66 (80)	71 (86)	137 (81)
Other ethnic group	1 (1)	0 (0)	0 (0)	1 (1)	1 (1)
Percentage trying to lose weight, n (%)	78 (98)	84 (94)	82 (97)	80 (95)	162 (96)
Percentage trying to avoid weight gain, n (%)	2 (3)	5 (6)	3 (4)	4 (5)	7 (4)
SSRQ^g^ score, mean (SD)	34.3 (6.8)	35.3 (5.8)	35.3 (6.5)	34.3 (6.1)	34.8 (6.3)
NCS^h^ score, mean (SD)	3.4 (0.8)	3.6 (0.7)	3.6 (0.8)	3.5 (0.8)	3.5 (0.8)

a n=79 due to missing data.

b n=84 due to missing data.

c n=168 due to missing data.

d GCSE: General Certificate of Secondary Education.

e BTEC: Business and Technology Education Council.

f n=79, 87, 83, 83, 166, respectively due to missing data.

g SSRQ: Short-form Self-Regulation Questionnaire.

h NCS: Need for Cognition Scale.

### Main Analyses

#### Effect of Information Length on Adherence

Mean adherence to the strategy was 74% (SD 25%) in the short information group and 69% (SD 23%) in the long information group. This difference was not statistically significant (*b*=−5.70, SE 3.74, 95% CI −13.00 to 1.77, *P*=.13); however, Cohen *d* indicated a small effect size (0.24). There was no significant moderation by need for cognition (*b*=5.50, SE 4.72, 95% CI −3.82 to 14.82, *P*=.25).

#### Effect of Implementation Intentions on Adherence

**Figure 2.**
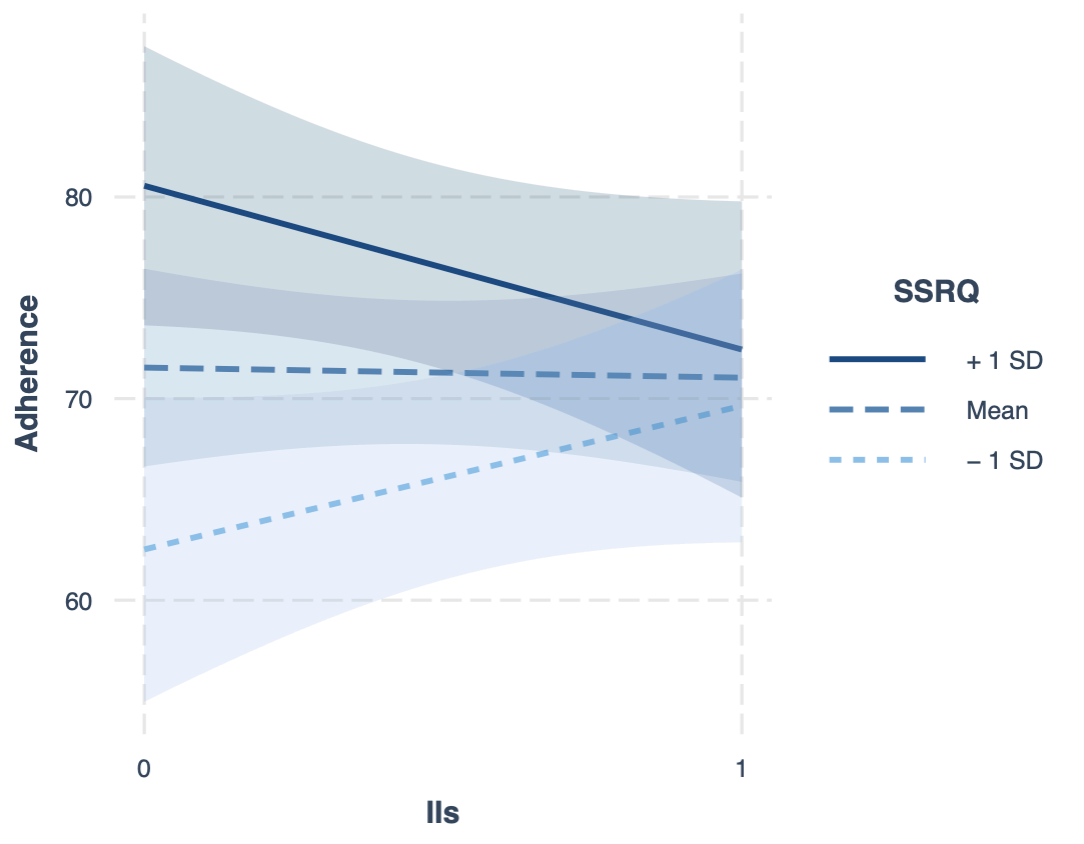
Simple slopes for the moderation effect of planning skills on the association between implementation intentions and adherence. II: implementation intention (0 absent, 1 present); SSRQ: Short-form Self-regulation Questionnaire.

Mean adherence to the strategy was 71% (SD 27%) in the implementation intentions group and 72% (SD 21%) in the group who received tips on strategy use. Contrary to predictions, there was no significant association between forming implementation intentions and strategy adherence (*b*=−1.34, SE 3.71, 95% CI −8.61 to 5.84, *P*=.73) but as predicted, there was a significant interaction between implementation intentions and SSRQ (*b*=−1.21, SE 0.58, 95% CI −2.34 to −0.07, *P*=.04). The Johnson-Neyman technique (Multimedia Appendix 5) revealed that implementation intentions (as opposed to tips) significantly increased adherence among those who scored below 13.01 on the SSRQ (poorer planners); however, this only represented 0.6% (1/169) of the sample. In addition to our predictions, implementation intentions decreased adherence among those who scored above 48.88 (skilled planners), but again, this only represented 0.6% (1/169) of the sample. This was further explored with simple slopes analysis, which revealed a similar pattern (Figure 2); implementation intentions increased adherence among participants with poor planning skills and decreased adherence among participants with good planning skills. These results suggest that planning skills may moderate the effect of implementation intentions on adherence, but the effect may be small.

#### Effect of Need for Cognition on Preference for Information Length

Contrary to predictions, there was no significant association between NCS score and odds of preference for shorter information length (odds ratio [OR] 0.96, 95% CI 0.45‐2.05, *P*=.92).

#### Effect of Implementation Intentions on Automaticity

Also contrary to predictions, there was no significant difference in SRBAI scores between participants who formed implementation intentions (mean 17.0, SD 7.1) and those who were given tips (mean 17.4, SD 6.6; *t*_138_=0.35, *P*=.73, 2-tailed).

### Additional Exploratory Analyses

#### Alternative Need for Cognition Measure

As expected, there was a significant positive correlation between the two need for cognition measures (*r_pb_*(198)=0.19, *P*=.01). Participants who reported that they like to understand the reasoning behind doctors’ advice (n=155) had a greater NCS score (mean 3.6, SD 0.8) than those who reported that they are happy to simply take doctors’ advice (n=45, mean 3.2, SD 0.8).

Contrary to the confirmatory analyses, the ANOVA exploring the moderating effect of the alternative need for cognition measure revealed a significant main effect of information length on adherence; adherence was greater in the short format group (mean 74%, SD 25%) than the long format group (mean 69%, SD 23%), *F*_1,165_=4.97, *P*=.03, η_p_^2^=0.03. For individuals with a low need for cognition (n=37), mean adherence was 83% (SD 16%) in the short format group and 66% (SD 29) in the long format group. For individuals with a high need for cognition (n=132), mean adherence was 72% (SD 26%) in the short format group and 69% (SD 21%) in the long format group. These figures are in line with expectations; however, the interaction effect between information length and the alternative need for cognition measure was not significant, *F*_1,165_=2.61, *P*=.11, η_p_^2^=0.02.

#### Alternative Planning Skills Measure

In addition, as expected, there was a significant positive correlation between the 2 planning skills measures, *r_pb_*(198)=0.58, *P*<.001. Participants who reported that they were good at making plans (n=84) had a greater SSRQ score (mean 38.8, SD 5.4) than those who reported that they struggled to make plans (n=116, mean 31.5, SD 5.0).

Participants who reported being good at making plans also reported significantly greater adherence (mean 76%, SD 25%) than those who struggled to make plans (mean 68%, SD 23%), *F*_1,165_=3.97, *P*=.048, η_p_^2^=0.02. For individuals with good planning skills (n=74), mean adherence was 71% (SD 30%) in the implementation intentions group and 81% (SD 17%) in the tips group. For individuals with poor planning skills (n=95), mean adherence was 71% (SD 24%) in the implementation intentions group and 66% (SD 22%) in the tips group. The interaction between implementation intentions and the alternative planning skills measure was significant, *F*_1,165_=4.66, *P*=.03, η_p_^2^=.03. However, 2-tailed independent samples *t* tests revealed no significant effect of implementation intentions on adherence in individuals with good planning skills (*t*_72_=1.80, *P*=.09) or individuals with poor planning skills (*t*_93_=−1.19, *P*=.25).

#### Effect of Strategy Content on Adherence

Mean strategy adherence was 72% (SD 28%) for sensory eating, 68% (SD 25%) for attending to fullness, 61% (SD 30%) for vegetables first, and 70% (SD 29%) for physical activity. There was no significant difference between the 4 strategies (*F*_3,196_=1.53, *P*=.21). The pattern of results remained the same when participants with less than 7 days of data were excluded (n=169).

#### Effect of Information Length on Self-Reported Ease of Understanding and Remembering

There was no significant association between information length and self-reported ease of understanding the strategy information (*b*=−2.62, SE 2.01, 95% CI −6.77 to 1.41, *P*=.22) and no significant interaction between information length and NCS score (*b*=0.72, SE 2.80, 95% CI −4.81 to 6.25, *P*=.80). However, ease of remembering was significantly higher among participants in the short format group (mean 83%, SD 22%) compared with those in the long format group (mean 74%, SD 25%; *b*=−9.08, SE 3.98, 95% CI −16.83 to −1.14, *P*=.02). There was no evidence for a moderation effect of NCS score (*b*=−0.84, SE 5.33, 95% CI −11.39 to 9.71, *P*=.87).

#### Effect of Information Length on Memory for the Strategy

Participants in the long format group were more likely to correctly remember the rationale for their assigned strategy than those in the short format group (OR 3.08, 95% CI 1.18‐8.05, *P*=.02). There was no moderation by NCS score (OR 0.81, 95% CI 0.22‐2.94, *P*=.75). Refer to Multimedia Appendix 6 for details of additional exploratory analyses.

#### Sensitivity Analyses

The analyses using the adherence variable were repeated using data from all 200 participants, where adherence was calculated over the full 2-week period (missing data were replaced with “non-adherent”). The pattern of results for all analyses remained unchanged apart from the ANOVA model exploring the moderating effect of the alternative need for cognition measure, where the main effect of information length became nonsignificant (*P*=.47). Refer to Multimedia Appendix 6 for details on additional exploratory analyses.

#### Qualitative Analyses

A total of 120 participants completed the qualitative survey at the end of the study. A summary of participant characteristics and full details of the analyses are provided in Multimedia Appendix 4. Responses generally centered around 4 key themes: app usability, strategy practicality, perceived benefits and effectiveness, and discussion of personal barriers to strategy use. The Avicenna Research app was generally well-received, with participants finding it easy to use and appreciating the daily survey notifications. However, many noted that reminders to use their assigned strategy would have been helpful. Most participants found their strategy easy to use, though many struggled to remember to apply it. Participants reported improvements in eating habits, physical activity, and overall well-being, and a small number of participants also reported weight loss. Family and work responsibilities often interfered with strategy use, along with other challenges such as health issues, travel, and financial constraints.

## Discussion

### Principal Findings

The novel key aim of this study was to explore whether adherence to brief weight management strategies over a 2-week period could be enhanced by manipulating information length, and whether this varied for individuals with different levels of need for cognition. The findings revealed no significant effect of information length on strategy adherence; however, the observed means were in the expected direction, with adherence 5 percentage points higher among those who viewed the shorter information. This represented a small effect size, which the study had not been powered to detect; while the planned regression analyses revealed a nonsignificant effect, an ANOVA, conducted as part of additional exploratory analyses, showed it as significant (*P*=.048). Because digital health interventions are often delivered to large numbers, an effect of this size may still be clinically significant. As such, these results warrant further exploration with an appropriately powered sample. More pronounced effects may also emerge over a longer intervention period.

Contrary to the findings of Williams-Piehota et al [16], the study failed to show that the effect of information length on adherence was influenced by need for cognition. Indeed, the overall pattern of results seems to suggest that even those with a high need for cognition may benefit from shorter information. In line with this finding, the need for cognition also failed to predict preference for information length. Findings from the exploratory analyses provide some insights into why shorter information may lead to better adherence; although memory for strategy rationale was better among those given the longer information, those given the shorter information reported that it was easier to remember the strategy. Thus, it is possible that shorter information leads to greater adherence simply because it helps people remember the strategy. A key implication of these findings is that digital health interventions may enhance adherence by limiting information length to no more than 100 words of action-oriented text. Optional links to additional details could then be provided for those who would prefer extra information.

In contrast to ease of remembering, ease of understanding was relatively high across both the short and long information groups (93% and 90%, respectively). It was also not influenced by the need for cognition, suggesting that need for cognition did not impact engagement with strategy content. It is possible that strategy understanding is better predicted by other participant characteristics, such as health literacy and cognitive ability. Health literacy encompasses skills in understanding and applying information about health issues [46], and higher levels have been associated with better health behaviors [47]. Likewise, engagement in health-promoting behaviors has been associated with greater cognitive ability as measured by general intelligence [48], processing speed [49], and analytic reasoning [50]. Given these correlations, it is possible that participants in this study had relatively high health literacy skills and cognitive abilities, which could have contributed to the high-reported ease of understanding, regardless of information length or need for cognition level. However, without direct measures of health literacy and cognitive ability, these interpretations remain speculative. Future studies may consider incorporating these measures in addition to the need for cognition.

The study’s second aim was to examine whether adherence could be enhanced with implementation intentions, and whether this varied for individuals with different levels of planning skills. In contrast to previous research [22-26], we found no evidence for the benefits of implementation intentions. This discrepancy may be due to our use of an active control condition that also communicated possible cue-action links. In a systematic review by Adriaanse et al [22], most studies used a passive control group where participants received no instructions or considerably fewer instructions than the experimental group. Effects of implementation intentions were stronger across studies with these weaker control groups compared with studies with active control conditions, which administered identical instructions to both control and experimental groups (apart from the manipulation). In these latter studies, the active control condition itself may promote goal achievement to some extent, thus reducing (but not entirely eliminating) the relative advantage of implementation intentions. As in our study, they may also sometimes specify cue-action links, further reducing their advantage. Thus, while implementation intentions may be effective in promoting goal achievement, other types of behavioral advice that specify cue-action links may be just as effective.

Furthermore, contrary to predictions, the study failed to find evidence to support the notion that implementation intentions achieve their effect by increasing automaticity. Automaticity of the strategies over the 2-week period did not differ significantly between those who formed implementation intentions and those who were given “tips,” with both groups reporting high automaticity (17.0 and 17.4 out of 20, respectively). Although it is plausible that the 2-week duration of the study was not sufficient to allow for any noticeable differences in automaticity to manifest, the high scores suggest that the “tips” were just as effective as implementation intentions in promoting automaticity. Again, this may be due to the inclusion of cue-action links in the “tips” condition. Further research with an additional control group would be needed to confirm this.

Nevertheless, in line with our predictions and with previous research [35], we did find that individuals with poorer planning skills reported greater adherence when given implementation intentions instead of “tips.” This finding highlights the potential for digital health interventions to enhance outcomes by encouraging the use of implementation intentions in individuals with poor self-regulation and planning ability. Unexpectedly, we also found that those with better planning skills reported greater adherence when provided with “tips” rather than implementation intentions. One possible explanation for this finding is that by asking good planners to form implementation intentions, we prevented them from using their own well-rehearsed, more flexible implementation strategies. Nevertheless, the moderation effect size was relatively small. Given our sample comprised weight management program patients who had volunteered to take part in our study, their motivation to try the strategies we provided them with may have been higher than the whole cohort of weight management program patients. As such, larger effect sizes may emerge across the whole population or over longer time frames, during which motivation may wane. The next key step in evaluating the effectiveness of personalized interventions based on planning skills would be to test this type of tailored content directly within a digital weight management program.

Another aim of the study was to explore whether simplified and more user-friendly versions of established measures of need for cognition and planning skills were effective substitutes. As expected, the short-form NCS was positively correlated with the alternative need for cognition measure (*r*=0.19), and the SSRQ goal setting subscale was positively correlated with the alternative planning skills measure (*r*=0.58). Furthermore, the alternative measures produced similar results to the standardized measures when examining their moderating effects on the influence of information length and implementation intentions on adherence. These findings are key, as both the short-form NCS and SSRQ goal-setting subscales consist of several questions (6 and 10, respectively) and are more burdensome to complete than our alternative measures, which consist of 1 question each, yet both measures yield similar results. The use of these brief alternative measures could facilitate personalized intervention by digital health companies based on need for cognition and planning ability, since they make it easier to capture differences in these measures among individuals.

### Strengths and Limitations

A key strength of this study is the sample, as participants were all patients who had been referred to a digital weight management program. They are therefore representative of the types of participants who would typically be targeted by this type of intervention. However, a key limitation is that participants had already completed the weight management program and thus may have been more motivated and had more practice at implementing behavioral advice. As participants were recruited at the end of the program, the sample we obtained is likely to represent individuals with higher adherence rates, since individuals with poor adherence may have dropped out before completing the program. This is evident in the high adherence levels observed across both mental and physical strategies during the 2-week period, despite the different levels of effort and skill required for these 2 strategy types. As noted above, significant differences may have emerged with an alternative sample, such as patients at the start of a weight management program, due to differences in motivation level and experience. It is therefore recommended to further investigate the effects of information length and implementation intentions within real-world digital weight management programs. These initiatives would enhance our understanding of what fosters adherence, enabling the development of more effective interventions.

### Conclusions

In conclusion, participants’ adherence to brief weight management strategies over a 2-week period appeared to be greater with shorter information compared with longer information. However, the study was not adequately powered to detect the observed small effect size. This effect was not moderated by need for cognition, suggesting that shorter information may be effective for both individuals with low and high need for cognition. There was no evidence that implementation intentions improved adherence compared with the same advice presented in the form of tips. There was some evidence suggesting that implementation intentions enhanced adherence for individuals with poorer planning skills, while the use of tips improved adherence in skilled planners, highlighting the need for personalization of behavior change interventions. The study demonstrated that simplified versions of standardized measures of need for cognition and planning skills may potentially be used as suitable substitutes, offering the practitioners tools to assess user characteristics more easily. While these findings offer valuable insights, they are preliminary and necessitate replication in future research.

## Supplementary material

10.2196/65260 Multimedia Appendix 1 Study consent.

10.2196/65260 Multimedia Appendix 2 Full strategy information.

10.2196/65260 Multimedia Appendix 3 Multiple choice questions.

10.2196/65260 Multimedia Appendix 4 Qualitative analyses.

10.2196/65260 Multimedia Appendix 5 Johnson-Neyman plot.

10.2196/65260 Multimedia Appendix 6 Additional analyses.

## Abbreviations

A-level: Advanced level
BTEC: Business and Technology Education Council
GCSE: General Certificate of Secondary Education
NCS: Need for Cognition Scale
OR: odds ratio
SRBAI: Self-report Behavioral Automaticity Index
SSRQ: Short-form Self-Regulation Questionnaire

## References

[1] Chao AM, Quigley KM, Wadden TA (2021). Dietary interventions for obesity: clinical and mechanistic findings. J Clin Invest.

[2] Hartmann-Boyce J, Johns DJ, Jebb SA, Aveyard P, Behavioural Weight Management Review Group (2014). Effect of behavioural techniques and delivery mode on effectiveness of weight management: systematic review, meta-analysis and meta-regression. Obes Rev.

[3] Carrière K, Khoury B, Günak MM, Knäuper B (2018). Mindfulness-based interventions for weight loss: a systematic review and meta-analysis. Obes Rev.

[4] Welton S, Minty R, O’Driscoll T (2020). Intermittent fasting and weight loss: systematic review. Can Fam Physician.

[5] Twells LK, Harris Walsh K, Blackmore A (2021). Nonsurgical weight loss interventions: a systematic review of systematic reviews and meta-analyses. Obes Rev.

[6] Lemstra M, Bird Y, Nwankwo C, Rogers M, Moraros J (2016). Weight loss intervention adherence and factors promoting adherence: a meta-analysis. Patient Prefer Adherence.

[7] Birch JM, Jones RA, Mueller J (2022). A systematic review of inequalities in the uptake of, adherence to, and effectiveness of behavioral weight management interventions in adults. Obes Rev.

[8] Rimal RN, Lapinski MK (2009). Why health communication is important in public health. Bull World Health Organ.

[9] Ngigi S, Busolo DN (2018). Behaviour change communication in health promotion: appropriate practices and promising approaches. ijird.

[10] Martin LR, DiMatteo MR (2014). The Oxford Handbook of Health Communication, Behavior Change, and Treatment Adherence.

[11] Cohen RA (2014). The Neuropsychology of Attention.

[12] Carr N (2020). The Shallows: What the Internet Is Doing to Our Brains.

[13] Yeykelis L, Cummings JJ, Reeves B (2014). Multitasking on a single device: arousal and the frequency, anticipation, and prediction of switching between media content on a computer. J Commun.

[14] Uncapher MR, Wagner AD (2018). Minds and brains of media multitaskers: current findings and future directions. Proc Natl Acad Sci U S A.

[15] Cacioppo JT, Petty RE (1982). The need for cognition. J Pers Soc Psychol.

[16] Williams-Piehota P, Schneider TR, Pizarro J, Mowad L, Salovey P (2003). Matching health messages to information-processing styles: need for cognition and mammography utilization. Health Commun.

[17] Sheeran P (2002). Intention—behavior relations: a conceptual and empirical review. Eur Rev Soc Psychol.

[18] Heckhausen H, Gollwitzer PM (1987). Thought contents and cognitive functioning in motivational versus volitional states of mind. Motiv Emot.

[19] Gollwitzer PM (2012). Handbook of Theories of Social Psychology.

[20] Gollwitzer PM (1999). Implementation intentions: strong effects of simple plans. Am Psychol.

[21] Gollwitzer PM, Sheeran P (2006). Adv Exp Soc Psychol.

[22] Adriaanse MA, Vinkers CDW, De Ridder DTD, Hox JJ, De Wit JBF (2011). Do implementation intentions help to eat a healthy diet? A systematic review and meta-analysis of the empirical evidence. Appetite.

[23] Carrero I, Vilà I, Redondo R (2019). What makes implementation intention interventions effective for promoting healthy eating behaviours? A meta-regression. Appetite.

[24] Bélanger-Gravel A (2013). A meta-analytic review of the effect of implementation intentions on physical activity. Health Psychol Rev.

[25] Silva M da, São-João TM, Brizon VC, Franco DH, Mialhe FL (2018). Impact of implementation intentions on physical activity practice in adults: a systematic review and meta-analysis of randomized clinical trials. PLoS ONE.

[26] Kompf J (2020). Implementation intentions for exercise and physical activity: who do they work for? A systematic review. J Phys Act Health.

[27] Hagerman CJ, Hoffman RK, Vaylay S, Dodge T (2021). Implementation intentions to reduce smoking: a systematic review of the literature. Nicotine Tob Res.

[28] Luszczynska A, Sobczyk A, Abraham C (2007). Planning to lose weight: randomized controlled trial of an implementation intention prompt to enhance weight reduction among overweight and obese women. Health Psychol.

[29] Armitage CJ, Norman P, Noor M, Alganem S, Arden MA (2014). Evidence that a very brief psychological intervention boosts weight loss in a weight loss program. Behav Ther.

[30] Benyamini Y, Geron R, Steinberg DM, Medini N, Valinsky L, Endevelt R (2013). A structured intentions and action-planning intervention improves weight loss outcomes in a group weight loss program. Am J Health Promot.

[31] Hayes JF, Balantekin KN, Graham AK, Strube MJ, Bickel WK, Wilfley DE (2021). Implementation intentions for weight loss in college students with overweight and obesity: a proof-of-concept randomized controlled trial. Transl Behav Med.

[32] Knäuper B, Shireen H, Carrière K (2020). The effects of if-then plans on weight loss: results of the 24-month follow-up of the McGill CHIP Healthy Weight Program randomized controlled trial. Trials.

[33] Verhoeven AAC, Adriaanse MA, de Ridder DTD, de Vet E, Fennis BM (2013). Less is more: the effect of multiple implementation intentions targeting unhealthy snacking habits. Euro J Social Psych.

[34] de Vet E, Oenema A, Brug J (2011). More or better: do the number and specificity of implementation intentions matter in increasing physical activity?. Psychol Sport Exerc.

[35] Allan JL, Sniehotta FF, Johnston M (2013). The best laid plans: planning skill determines the effectiveness of action plans and implementation intentions. Ann Behav Med.

[36] Ahmadyar K, Szypula J, Bogosian A, Tapper K (2023). Improving adherence to weight management strategies: information length and implementation intentions. OSF.

[37] Seguias L, Tapper K (2018). The effect of mindful eating on subsequent intake of a high calorie snack. Appetite.

[38] Jordan CH, Wang W, Donatoni L, Meier BP (2014). Mindful eating: trait and state mindfulness predict healthier eating behavior. Pers Individ Dif.

[39] Nishino K, Sakurai M, Takeshita Y, Takamura T (2018). Consuming carbohydrates after meat or vegetables lowers postprandial excursions of glucose and insulin in nondiabetic subjects. J Nutr Sci Vitaminol (Tokyo).

[40] Buffey AJ, Herring MP, Langley CK, Donnelly AE, Carson BP (2022). The acute effects of interrupting prolonged sitting time in adults with standing and light-intensity walking on biomarkers of cardiometabolic health in adults: a systematic review and meta-analysis. Sports Med.

[41] Patient-centric clinical trial and health research software platform. Avicenna Research.

[42] Neal DJ, Carey KB (2005). A follow-up psychometric analysis of the self-regulation questionnaire. Psychol Addict Behav.

[43] Lins de Holanda Coelho G, H P Hanel P, J Wolf L (2020). The very efficient assessment of need for cognition: developing a six-item version. Assessment.

[44] Gardner B, Abraham C, Lally P, de Bruijn GJ (2012). Towards parsimony in habit measurement: testing the convergent and predictive validity of an automaticity subscale of the Self-Report Habit Index. Int J Behav Nutr Phys Act.

[45] Hayes AF (2017). Introduction to Mediation, Moderation, and Conditional Process Analysis: A Regression-Based Approach.

[46] Ishikawa H, Kiuchi T (2010). Health literacy and health communication. Biopsychosoc Med.

[47] Šulinskaitė K, Zagurskienė D, Blaževičienė A (2022). Patients’ health literacy and health behaviour assessment in primary health care: evidence from a cross-sectional survey. BMC Prim Care.

[48] Auld MC, Sidhu N (2005). Schooling, cognitive ability and health. Health Econ.

[49] Anstey KJ, Low LF, Christensen H, Sachdev P (2009). Level of cognitive performance as a correlate and predictor of health behaviors that protect against cognitive decline in late life: the path through life study. Intelligence.

[50] Junger M, van Kampen M (2010). Cognitive ability and self-control in relation to dietary habits, physical activity and bodyweight in adolescents. Int J Behav Nutr Phys Act.

