# Supplementary material for: Effects of Information Length and Implementation Intentions on Adherence to Weight Management Strategies: Experimental Study
Source: JMIR Mhealth Uhealth. 2025 Aug 8;13:e65260. doi: 10.2196/65260 (PMC12334108; doi:10.2196/65260)
Supplement: Multimedia Appendix 1 [file mhealth-v13-e65260-s001.pdf]

Default Question Block

Use of a brief weight management strategy

Thank you for your interest in taking part in this study. Please click the link below to review the study information. You can also download the document to review later.

Study Information

To assess whether you are eligible to participate in this study, please answer the questions that follow.

Block 2

Are you aged 18 years or older?

- ○ Yes
- ○ No

Are you interested in losing weight or avoiding weight gain?

- ○ Yes
- ○ No

Do you have access to a smartphone?

- Yes
- No

Are you following a meal replacement diet (i.e. you consume low-calorie shakes instead of a meal)?

- Yes
- No

What is your weight in kilograms? Please enter numbers only.

If you don't know your weight in kilograms, please click here to use the weight converter.

What is your height in centimetres? Please enter numbers only.

If you don't know your height in centimetres, please click here to use the height converter.

Not eligible

Thank you for completing this survey. Unfortunately, you are not eligible to take part in this study. We would like to thank you for your interest though. Please feel free to contact Khaleda at Khaleda.Ahmadyar.1@city.ac.uk if you have any

questions.

Eligible

Use of a brief weight management strategy

Thank you for completing this survey. We're happy to tell you that you're eligible to take part in this study!

Please click the link below to review the study information. You can also download the document to review later.

Study Information

If you would like to take part in the study, please confirm the following:

- ☐ I confirm that I have read and understood the study information dated 30/07/2023, (Version 1) for the above study. I have had the opportunity to consider the information and ask questions which have been answered satisfactorily.
- ☐ I understand that my participation involves downloading a smartphone application, learning about a weight management strategy, and completing surveys.
- ☐ I understand that my participation is voluntary and that I am free to withdraw without giving a reason and without being penalised or disadvantaged.
- ☐ I understand that I will be able to withdraw my data up to the point at which I complete the follow-up survey.
- ☐ I agree to City recording and processing this information about me. I understand that this information will be used only for the purpose(s) explained in the participant information and my consent is conditional on City complying with its duties and obligations under the General Data Protection Regulation (GDPR).
- ☐ I understand that the anonymised data will also be made available to others on the Open Science Framework to allow them to verify or follow-up on the results.
- ☐ I agree to take part in the above study.

Alternatively, if you have decided not to take part, please close your browser

window now.

Ethica instructions

To enrol onto the study, you will need to download a smartphone application called ‘Ethica’ and sign up for an account. Instructions on how to do this will be provided on the next page.

Please enter your email address so we can email you the instructions in case you need them later. ${q://QID17/ChoiceTextEntryValue}

Please confirm your email address

Powered by Qualtrics
