# Supplementary material for: Effects of Information Length and Implementation Intentions on Adherence to Weight Management Strategies: Experimental Study
Source: JMIR Mhealth Uhealth. 2025 Aug 8;13:e65260. doi: 10.2196/65260 (PMC12334108; doi:10.2196/65260)
Supplement: Multimedia Appendix 2 [file mhealth-v13-e65260-s002.docx]

**Appendix 1.** Strategy information.

**STRATEGY 1: SENSORY EATING (SHORT)**

**Pay attention to the taste and texture of food in your mouth**

People who eat more slowly, eat less. And the longer food spends in your mouth, the more it promotes the release of gut hormones that help you feel full. To slow your eating, pay attention to the sensory properties of your food - its taste, texture, and temperature. Notice how these change as you chew. Imagine you are a culinary critic, trying to describe the food you’re eating.

**STRATEGY 1: SENSORY EATING (LONG)**

**How to slow down your eating**

The speed at which you eat tends to vary depending on the situation. For example, you may eat faster when you’re very hungry, when you’re in a hurry, or simply when you’re enjoying a really good food! However, eating rate also varies between individuals with some people being naturally fast eaters and others being inclined to eat more slowly. Those who do eat more slowly tend to eat less and are less likely to struggle with their weight.

There are several reasons why slower eating could help you eat less. First, it can take around 20 minutes for fullness signals from your stomach to register in your brain. This means that if you eat very quickly you could overeat before you realise it. You may have experienced this yourself, when you’ve been very hungry, eaten a very large portion and perhaps regretted it later when your stomach felt uncomfortably full. Eating more slowly allows you to monitor more accurately how full you’re getting, which may help prevent you from overeating.

The body also responds to food in the mouth by releasing hormones that help you feel full. The longer food spends in the mouth, the greater the release of these hormones. This means that chewing your food more slowly or chewing it for longer may make you feel fuller - this in turn could help you feel more satisfied with smaller servings.

Additionally, as you eat a food, your enjoyment of that food goes down relative to other foods with contrasting tastes. This is why you can sometimes feel like you’ve ‘had enough’ of a savoury main course but still have room for dessert! Eating a food more slowly may mean you reach this point of having ‘had enough’ after a smaller serving.

Slower eating has a couple of other advantages too – it can improve digestion and help your body absorb more nutrients from your food.

So, how can you slow down your rate of eating? There are plenty of suggestions out there. Some experts recommend chewing every mouthful for 30 seconds or putting your cutlery down between each bite. Others propose counting the number of times you chew each bite then trying to double this number. You can even buy an electronic fork that will beep at you if you’re eating too fast! However, those who try these strategies don’t always enjoy them, which means they can give up on them quite quickly.

An alternative strategy is to focus on the sensory properties of your food as you eat. This may be an easier strategy to stick to as it can actually increase the amount of pleasure you get from your food.

How can you go about doing this? Start by simply noticing the food in your mouth before you bite into it. Explore the feel and taste of it on your tongue and against the roof of your mouth. Is it smooth or textured? Sweet or salty? Sour or bitter? Warm or cool? Does the food taste different in different parts of your mouth?

As you bite into your food, notice any change in these sensations. Are new tastes released? What flavours can you detect? Is there anything surprising about the texture, or was it how you imagined it would be? Does your food make a sound?

Continue to notice these things as you chew. How do the tastes and textures change over time? Are there many different flavours or just one or two? Do new flavours emerge as the food breaks down? Do they get stronger or weaker?

You might find it helpful to imagine you are a culinary critic, who has been asked to comment on the food you’re eating. How would you describe it to others? Is the texture hard or soft? Crumbly or crisp? What do the tastes and flavours remind you of? As sharp and lemony as a Spring morning? Or with hints of cinnamon that bring memories of Christmas? Which words and phrases best capture your experience? Feel free to be as poetic as you like!

**STRATEGY 2: ATTENDING TO FULLNESS (SHORT)**

**Pay attention to your fullness**

Feelings of fullness can prompt you to stop eating. However, sometimes it’s easy to ignore these signals, especially if your attention is elsewhere. When you eat, try to notice how your body feels, especially your stomach. If you’re doing something else, like watching TV or using your phone, keep asking yourself if you’re still hungry or if you’re feeling satisfied. If you’re satisfied, stop eating and put any remaining food out of reach.

**STRATEGY 2: ATTENDING TO FULLNESS (LONG)**

**How to avoid overeating**

Eating too much in one sitting can cause your stomach to stretch beyond its normal size, making you feel uncomfortable. Unsurprisingly, this type of overeating can lead to weight gain. However, even eating just a little more than you need can lead to weight gain if it’s something you do regularly. For example, eating an extra 100 calories a day could lead to weight gain of 10 lb (4.5 kg) by the end of the year. It’s easy to eat an extra 100 calories without really noticing it, for example by having an extra slice of bread, chunk of cheese or scoop of ice cream.

There are lots of reasons why you may eat more than you need. Sometimes it might simply be that you’re really enjoying the taste of the food! However, at other times you may eat too much because you’re reluctant to waste food or you’ve simply got into the habit of eating everything on your plate. Large servings in restaurants and other food outlets don’t help, as the more food you’re served, the more you’re likely to eat.

Another important contributor to overeating is environmental distractions. People tend to eat more when they’re doing other things such as watching TV, chatting or using a smartphone. For example, one study found that people ate 15% more when distracted by a smartphone! This happens because you can only pay attention to one thing at a time, so if you’re engrossed in a TV show or conversation, you can end up eating on autopilot and fail to notice when you’ve had enough or when you’re no longer really enjoying the food. As a result, you may eat much more than you want or need.

So, how can you avoid overeating? Serving yourself smaller portions is a good place to start. You could also try eating without distractions, for example by turning off your phone or TV. However, this will not always be practical or desirable. Sometimes you may need to multi-task to get things done and other times you may get a lot of pleasure from pairing food with conversation or entertainment.

An alternative strategy is to cultivate the habit of periodically bringing your attention back to your food and your body, to monitor your feelings of hunger and fullness. By repeatedly bringing your awareness to your body as you eat, you’re more likely to spot the point at which you’ve eaten enough. In this way, you can avoid eating too much.

To become more aware of sensations of fullness, keep pausing to check in with your body. Stop eating for a moment, put down your utensils and pay attention to how hungry or satisfied you feel. Are you still truly hungry, or are you starting to feel full? Take a moment to assess how full you are and then consciously decide whether or not you want to keep eating. If you feel you’ve eaten enough, put any remaining food out of reach.

This awareness will allow you to adjust your portions and stop eating before you eat more than you need and before you reach the point of uncomfortable fullness. Remember, it's okay to leave food on your plate.

You may also find it helpful to use a hunger-fullness scale. Before and during your meals, assess your hunger and fullness levels on a scale from 1 to 10, with 1 being starving and 10 being uncomfortably stuffed. Aim to start eating when you're at a moderate level of hunger (around 3-4) and stop eating when you're comfortably satisfied (around 6-7). This can help you keep in touch with your body’s signals and avoid eating too much.

**STRATEGY 3: VEGETABLES FIRST (SHORT)**

**Eat fibre-rich vegetables or salad first**

Hunger and cravings may sometimes be the result of a dip in blood glucose levels. You may experience a dip in blood glucose after eating carbohydrate-rich foods that release glucose into the bloodstream very quickly. To slow glucose absorption, try eating fibre-rich vegetables or salad at the start of your meal. If you’re having a carbohydrate-based snack or breakfast, try grabbing a handful of salad to eat first.

**STRATEGY 3: VEGETABLES FIRST (LONG)**

**A tip for reducing hunger and cravings**

Hunger and cravings may sometimes be caused by low blood glucose levels. Blood glucose refers to the amount of glucose in your bloodstream. Glucose is a type of sugar and blood glucose levels are influenced by a range of different factors including the food you eat.

When you eat foods containing carbohydrates, this is broken down by the body into glucose and absorbed into the bloodstream. If you eat complex carbohydrates (like wholegrains) they take longer for your body to break down, and the glucose enters your bloodstream more slowly. However, other types of carbohydrates (such as refined sugars) result in glucose entering your bloodstream much more quickly.

When glucose enters your bloodstream quickly, it can lead to a rapid rise (or ‘spike’) in blood glucose levels. Your body responds by releasing lots of insulin in order to bring these levels down. This can in turn lead to a sharp drop (or ‘dip’) in blood glucose levels. It is this dip that could make you feel hungry and could lead you to crave sugary, high calorie foods.

If you find yourself experiencing hunger and cravings a few hours after eating, it may be because of a dip in your blood glucose levels. By keeping your blood glucose levels more stable, and avoiding large spikes and dips, you may be able to reduce hunger and cravings.

There may also be other benefits to keeping your blood glucose levels more stable. Blood glucose dips may be associated with feelings of tiredness, irritability, and low mood as well as difficulties with concentration. More stable blood glucose could therefore help improve your mood and concentration and help you feel more energised throughout the day.

So, how can you keep your blood glucose levels more stable when eating? There are a few things you can try. Instead of choosing refined carbohydrates like white bread, white rice, and white pasta, go for wholegrains like wholewheat bread, brown rice and brown pasta. These complex carbohydrates are digested more slowly, causing a slower rise in blood glucose. It’s also a good idea to try to limit highly processed foods that contain lots of refined sugars, such as biscuits, cakes, sweets and sugary drinks as these foods will release glucose into the bloodstream more quickly.

However, eating any kind of carbohydrate leads to an increase in blood glucose. And some people are more prone to blood glucose spikes and dips than others. Another way of reducing the impact of carbohydrates is to eat vegetables or salad first. Vegetables and salad are high in fibre which can slow down the absorption of glucose, preventing your blood glucose from rising too quickly.

Vegetables and salad also tend to have a high water content which makes them more filling. This means that if you eat them first, you may end up eating less of the high carbohydrate foods that could cause your blood glucose to spike and dip.

So, for more stable blood glucose levels, try to eat the salad or vegetables on your plate first, before eating the rest of your meal. If you’re having a carbohydrate-based snack or a breakfast that doesn’t contain vegetables, try to grab a handful of salad to eat first.

To make your vegetables even tastier, you could try different ways of cooking them, like steaming, sautéing, or roasting. You could also try them stir-fried or raw in salads. Different herbs, spices, and healthy dressings can add extra flavour whilst a squeeze of lemon or a sprinkle of vinegar can give them a tangy twist. So, next time you eat, start with your veggies!

**STRATEGY 4: INCREASE PHYSICAL ACTIVITY (SHORT)**

**Do 5 minutes of physical activity after eating**

Hunger and cravings may sometimes be the result of a dip in blood glucose levels. These dips can occur after carbohydrate-rich food has led to a rapid rise then fall in blood glucose. You can reduce this rise (and subsequent fall) by doing at least 5 minutes of physical activity after eating, since this makes your muscles use some of your blood glucose. This activity could take the form of a short walk, some resistance exercises or even just catching up on daily chores.

**STRATEGY 4: INCREASE PHYSICAL ACTIVITY (LONG)**

**A tip for reducing hunger and cravings**

Hunger and cravings may sometimes be caused by low blood glucose levels. Blood glucose refers to the amount of glucose in your bloodstream. Glucose is a type of sugar and blood glucose levels are influenced by a range of different factors including the food you eat.

When you eat foods containing carbohydrates, this is broken down by the body into glucose and absorbed into the bloodstream. If you eat complex carbohydrates (like wholegrains) they take longer for your body to break down, and the glucose enters your bloodstream more slowly. However, other types of carbohydrates (such as refined sugars) result in glucose entering your bloodstream much more quickly.

When glucose enters your bloodstream quickly, it can lead to a rapid rise (or ‘spike’) in blood glucose levels. Your body responds by releasing lots of insulin in order to bring these levels down. This can in turn lead to a sharp drop (or ‘dip’) in blood glucose levels. It is this dip that could make you feel hungry and could lead you to crave sugary, high calorie foods.

If you find yourself experiencing hunger and cravings a few hours after eating, it may be because of a dip in your blood glucose levels. By keeping your blood glucose levels more stable, and avoiding large spikes and dips, you may be able to reduce hunger and cravings.

There may also other benefits to keeping your blood glucose levels more stable. Blood glucose dips may be associated with feelings of tiredness, irritability, and low mood as well as difficulties with concentration. More stable blood glucose could therefore help improve your mood and concentration and help you feel more energised throughout the day.

So, how can you keep your blood glucose levels more stable when eating? There are a few things you can try. Instead of choosing refined carbohydrates like white bread, white rice, and white pasta, go for wholegrains like wholewheat bread, brown rice and brown pasta. These complex carbohydrates are digested more slowly, causing a slower rise in blood glucose. It’s also a good idea to try to limit highly processed foods that contain lots of refined sugars, such as biscuits, cakes, sweets and sugary drinks as these foods will release glucose into the bloodstream more quickly.

However, eating any kind of carbohydrate leads to an increase in blood glucose. And some people are more prone to blood glucose spikes and dips than others. Another way of reducing the impact of carbohydrates is to be physically active for at least 5 minutes after eating. This will make your muscles use some of your blood glucose for energy, which will in turn help reduce the amount your blood glucose rises. And if you can prevent your blood glucose from rising too high, you will reduce the size of any subsequent dip.

Aim to start your 5 minutes of physical activity as soon as possible after finishing your meal, though ideally within 30 minutes. The timing is important because it corresponds with when your blood glucose starts to rise.

Choose an activity that is practical, convenient, and enjoyable. It doesn’t have to be too intense - the important thing is that it gets your muscles moving. Walking is a good choice because you can do it anywhere, indoors or outdoors. Simple strength exercises like squats, sit-ups or lunges can work well too. Or you could just catch up on daily chores, such as tidying, cleaning or running errands – anything that gets you up on your feet for 5 minutes. If you have limited mobility, seated exercises or gentle stretching routines can be a good option. The key is to get your body moving and increase your heart rate. So, next time you eat, remember to also get moving!
