# Supplementary material for: Effects of Information Length and Implementation Intentions on Adherence to Weight Management Strategies: Experimental Study
Source: JMIR Mhealth Uhealth. 2025 Aug 8;13:e65260. doi: 10.2196/65260 (PMC12334108; doi:10.2196/65260)
Supplement: Multimedia Appendix 3 [file mhealth-v13-e65260-s003.docx]

**Appendix 2.** Multiple choice questions.

**Sensory eating**

1. Paying attention to the taste and texture of food as you eat:
   1. slows eating, which helps reduce the number of calories your body absorbs
   2. slows eating, which promotes the release of gut hormones that help you feel full
   3. stimulates the senses, which can lead you to eat more
   4. helps your body distinguish between healthier and less healthy foods

**Attending to fullness**

1. Attending to feelings of fullness during a meal:
   1. helps direct your attention toward healthier foods
   2. may distract you from making healthy food choices
   3. can help you stop eating when you’ve had enough
   4. may mean you prioritise fullness over healthiness

**Vegetables first**

1. Eating fibre-rich vegetables or salad at the start of the meal:
   1. provides specific vitamins and minerals that satisfy hunger and reduce cravings
   2. slows the absorption of glucose, reducing blood glucose dips
   3. triggers a hormone response that suppresses appetite
   4. can lead to carbohydrate cravings that make you more likely to snack

**Increase physical activity MCQ**

1. Light physical activity after eating:
   1. helps muscles use glucose, reducing subsequent blood glucose dips
   2. increases your awareness of fullness signals
   3. can distract the mind from food
   4. interferes with digestion
