# Supplementary material for: Effects of Information Length and Implementation Intentions on Adherence to Weight Management Strategies: Experimental Study
Source: JMIR Mhealth Uhealth. 2025 Aug 8;13:e65260. doi: 10.2196/65260 (PMC12334108; doi:10.2196/65260)
Supplement: Multimedia Appendix 4 [file mhealth-v13-e65260-s004.docx]

**Appendix 3.** Qualitative analyses.

**Method**

Four optional open-ended questions were administered on the study app at the end of the study: *“Please describe your experience of taking part in this study over the past 2 weeks. How did you find using the strategy? How did you feel about this as a strategy to help you manage your weight?”*, *“Was there anything you particularly liked about the strategy, or the way it was delivered? Which aspects (if any) worked well for you?”*, *“What challenges (if any) did you encounter when using the strategy? What made it difficult, or what prevented you from using the strategy?”* and *“How do you think the strategy, or the way it was delivered, could be improved?”.* The app enabled participants to either type or audio record their responses.

Data were analysed using content analysis on NVivo (version 12). Audio responses were transcribed and collated with written responses. Codes were then developed based on common responses. Data for each question were first coded separately, then condensed across all four questions by combining similar codes and developing overarching themes. A subset of responses (10%) was double coded, and the inter-observer reliability was 85%.

**Results**

A total of 120 participants completed the qualitative survey at the end of the study. A summary of participant characteristics is provided in Table A1. Four overarching themes were identified and are described below. See Table A2 for full details of the themes and subcategories.

***Theme 1: Delivery***

Many responses indicated that the Avicenna Research app was easy and simple to use and the daily survey notifications were useful. The strategy information was reported to be helpful, with many reporting that the rationale behind the strategy was explained well and was easy to understand. A small number of responses indicated the need for a longer timeframe to use the strategy in order to see benefits.

A common comment about the delivery of the study was the lack of reminders to use the strategy. The majority of responses indicated that notifications to remind participants to use their assigned strategy would have been helpful. The need for reminders was mentioned more in the two mindfulness conditions compared to the two physical strategies. This response was also more common among those who received tips than those who formed implementation intentions. A small number of responses indicated that more information about the assigned strategy would have been helpful, including both background information and more detail on using the strategy.

***Theme 2: Strategy content***

The majority of responses indicated that the assigned strategy was easy to use, and many reported that it was easy to get into the habit of using the strategy. This response was more common among those in the physical activity condition. The strategy content was reported to be helpful in many responses, particularly in relation to the ‘attending to fullness’ condition. About half of responses indicated that although the strategy was easy to use, it was difficult to remember to use it. This was more common among those in the two mindfulness conditions than the two physical conditions, and among those in the short format group than the long format group.

***Theme 3: Outcomes***

Many responses indicated that participants noticed an improvement in their health behaviours as a result of using their assigned strategy. The most common behaviours reported were improvements in eating habits, where participants noticed they were less prone to snacking and overeating, and more mindful of their food intake. Improvements in physical activity were also reported. Some responses indicated that using the assigned strategy helped with weight management. Other positive outcomes reported included feeling better and healthier and learning something new. A small number of responses indicated that participants had not noticed a change in their weight, or that they did not believe the strategy would help them with their weight management.

***Theme 4: Personal factors***

Some responses indicated that having a family interfered with use of the assigned strategy. This was often due to family responsibilities keeping participants too busy, thus having no time to use the strategy, or that their family distracted them and thus they were not able to focus on using the strategy. Work responsibilities were also reported to interfere with strategy use in some responses. Participants were either too busy with work to use the strategy, or the nature of their work did not allow them to use the strategy, e.g., they had no space to do physical activity, or they had no or short lunch breaks. Other interferences were also mentioned in some responses, such as having health issues, pet responsibilities, travelling, and financial issues.

**Discussion**

The qualitative aspect of the study revealed that comments about the study delivery were generally positive. Participants found the app simple and easy to use but reported that reminders would have been helpful. The assigned strategy was mostly reported to be helpful, effective, and easy to implement though there were some reports of finding it difficult to remember to use the strategy. There were reported improvements in health behaviours such as eating habits and physical activity as well as supporting weight management. Lack of adherence was commonly attributed to personal factors such as family or work commitments and other interferences such as health issues, financial issues and travelling. These findings provide useful insights on the acceptability of brief weight management interventions and highlight additional barriers which can be targeted to enhance adherence, such as difficulty remembering to use the strategy.

**Table A1.** Summary of participant characteristics who participated in the qualitative survey.

| Characteristic | Count |
| --- | --- |
| **Gender**  Woman  Man  Prefer not to say | 77  41  1 |
| **Age**  20-29  30-29  40-49  50-59  60-69  70-79 | 5  11  36  38  24  6 |
| **Education**  No formal education  GCSEs/O-levels or equivalent  BTEC or equivalent  A-levels or equivalent  Undergraduate degree or equivalent  Master’s degree or equivalent  Doctoral degree or equivalent  Prefer not to say | 3  23  16  10  45  21  1  1 |
| **Ethnicity**  Asian or Asian British  Black, African, Caribbean or black British  Mixed or multiple ethnic groups  White  Prefer not to say | 11  5  2  99  3 |
| **BMI**  25 – 29.9 (overweight)  >30 (obese) | 31  89 |
| **Strategy content**  Sensory eating  Fullness  Vegetables first  Physical activity | 27  34  28  31 |
| **Planning prompts**  Implementation intentions  Tips | 52  68 |
| **Information format**  Short  Long | 55  65 |

**Table A2.** Themes and subcategories identified in qualitative analysis.

| Themes | Subcategories | Responses |
| --- | --- | --- |
| **Delivery** | App features were useful | 52 |
|  | Strategy information was helpful | 26 |
|  | Need a longer timeframe | 10 |
|  | Need more app features | 41 |
|  | Need reminders to use strategy | 47 |
|  | Need more information about strategy | 10 |
| **Strategy content** | Easy to use strategy or form a habit | 103 |
|  | Strategy was helpful or effective | 68 |
|  | Difficult to use strategy or form a habit | 45 |
|  | Difficult to remember to use strategy | 67 |
|  | Strategy was not helpful or effective | 16 |
| **Outcomes** | Enjoyed the experience of study | 3 |
|  | Improved health behaviours | 68 |
|  | Helped with weight management | 15 |
|  | Helped feel better | 4 |
|  | Learnt something new | 6 |
|  | Did not help with weight management | 9 |
| **Personal factors** | Family | 10 |
|  | Work | 13 |
|  | Other interferences | 38 |
