# Supplementary material for: Effects of Information Length and Implementation Intentions on Adherence to Weight Management Strategies: Experimental Study
Source: JMIR Mhealth Uhealth. 2025 Aug 8;13:e65260. doi: 10.2196/65260 (PMC12334108; doi:10.2196/65260)
Supplement: Multimedia Appendix 5 [file mhealth-v13-e65260-s005.docx]

**Appendix 5.** Johnson-Neyman Plot for the moderation effect of planning skills on the association between implementation intentions and adherence.

IIs: implementation intentions; SSRQ: Short-form Self-Regulation Questionnaire.
