# Supplementary material for: Effects of Information Length and Implementation Intentions on Adherence to Weight Management Strategies: Experimental Study
Source: JMIR Mhealth Uhealth. 2025 Aug 8;13:e65260. doi: 10.2196/65260 (PMC12334108; doi:10.2196/65260)
Supplement: Multimedia Appendix 6 [file mhealth-v13-e65260-s006.docx]

**Appendix 4.** Additional analyses.

**Additional reporting for the moderating effect of SSRQ on association between implementation intentions and adherence**

There was a significant positive association between SSRQ scores and strategy adherence (*b*=2.63, SE 0.94, 95% CI 0.78 - 4.48, *P*=.01), suggesting that greater planning skills were associated with greater adherence.

**Additional exploratory analyses**

***Effect of free time on adherence***

Participants responded to *“Which of the following best describes you?”* with ‘I’m very busy, and never seem to have enough time for everything I need to do’ / ‘I’m quite busy, but if something unexpected comes up, I can usually make time to deal with it’ / ‘I typically have plenty of free time to spend how I choose’.

Mean strategy adherence was 67% (SD 25) among participants who stated they were *‘very busy’* (n=54), 73% (SD 24) among those who were *‘quite busy’* (n=86), and 77% (SD 21) among those who stated they had *‘plenty of free time’* (n=29). However, there was no significant main effect of free time on adherence (b=4.99, SE 2.64, 95% CI -.29 to 10.28, P=.06) and no moderation by information length (Ps>.05). There was no significant difference in adherence between the *‘very busy’* and *‘quite busy’* group (*b*=17.92, SE 13.47, 95% CI -8.68 to 44.52, *P*=.10) or the *‘plenty of free time’* group (*b*=10.71, SE 17.27, 95% CI -23.39 to 44.80, *P* = .54). The effect of information length on adherence did not significantly differ between the *‘very busy’* group and the *‘quite busy’* group (*b*=-8.59, SE 8.34, 95% CI -25.05 to 7.87, *P*=.30) or the *‘plenty of free time’* group. (*b*=0.65, SE 11.13, 95% CI -22.61 to 21.32, *P*=.95).

***Effect of diet/weight priority on adherence***

Participants were asked *“Thinking about all the things going on in your life right now, how much of a concern is your diet or weight?”* with response options of ‘It's the thing I'm most concerned about right now’ / ‘It's one of several important concerns I have right now’ / ‘There are other things I'm more concerned about right now’.

Mean adherence was 77% (SD 19) in individuals whose diet/weight was of high priority (n=48), 69% (SD 26) in those with medium priority (n=117) and 74% (SD 9) in those with low priority (n=4). The main effect of diet/weight priority on adherence approached significance (b=-6.20, SE 3.15, 95% CI -12.58 to -0.11, P=.054). There was no significant moderation by information length (Ps>.05). The analysis revealed no significant difference in adherence between the high priority group and medium priority group (*b*=-6.17, SE 12.64, 95% CI -31.13 to 18.78, *P*=.63) or the low priority group (*b*=-7.58, SE 50.85, 95% CI -108.00 to 92.83, *P*=.88). The effect of information length on adherence did not significantly differ between the high priority group and the medium priority group (*b*=-0.65, SE 8.33, 95% CI -17.10 to 15.80, *P*=.94) or the low priority group (*b*=3.66, SE 28.50, 95% CI -52.60 to 59.93, *P*=.90).

***Moderating effect of preference for time spending learning new things on the association between information format and adherence***

Participants were asked *“If you were part of a weight management programme, how much time would you prefer to spend learning new things?”* with response options ‘No more than a few minutes a day or 30 minutes a week’ / ‘Up to 15 minutes a day or 1 to 2 hours a week’ / ‘Up to 30 minutes a day or 3 to 4 hours a week’.

Preference for time spending learning new things did not moderate the impact of information length on strategy adherence (Ps>0.05). The impact of information length on strategy adherence did not significantly differ between participants who preferred to spend a few minutes a day/30 mins a week learning new things and those who preferred to spend 15 mins a day/1-2 hours a week (*b*=2.13, SE 9.12, 95% CI -15.85 to 20.11, *P*=.82) or those who preferred to spend 30 mins a day/2-4 hours a week (*b*=0.83, SE 11.43, 95% CI -21.75 to 23.41, *P*=.94).

***Moderating effect of information format on association between need for cognition and preference for information length***

Information format and the mean centred interaction between information length and NCS was added to the ordinal logistic regression model testing the effect of need for cognition on preference for information length. The interaction effect was not significant (OR=0.93, 95% CI 0.20 - 4.29, *P*=.93), suggesting that information length did not moderate the association between need for cognition and odds of preference for shorter information length.

***Reasons for non-adherence***

Additional descriptive analyses were conducted to explore whether non-adherence was a result of forgetting to use the strategy or for other reasons. The mean percentage of days that participants forgot to use the strategy was 16% (SD 20) and the mean percentage of days that participants did not use the strategy for another reason was 14% (SD 18) for those who completed at least 1 daily survey (n=195). For those who completed at least 7 daily surveys (n=169), the mean percentage of days participants forgot to use the strategy was 15% (SD 18) and the mean percentage of days they reported not using the strategy for another reason was 14% (SD 17). The data suggest that non-adherence was a result of both forgetting to use the strategy and other reasons.

***Strategy helpfulness ratings***

Participants responded to *“How helpful did you find the strategy?”* on a 5-point scale ranging from 1 (very unhelpful) to 5 (very helpful). There were no significant differences in helpfulness ratings across sensory eating (mean 3.8, SD 1.0), attending to fullness (mean 3.9, SD 0.9), vegetables first (mean 3.9, SD 1.1) and physical activity (mean 4.1, SD 0.9), F3,139=0.44, P=.73 (n=143 as not all participants used the strategy).
